# Amphiregulin and Epiregulin mRNA expression in primary colorectal cancer and corresponding liver metastases

**DOI:** 10.1186/1471-2407-12-88

**Published:** 2012-03-13

**Authors:** Hidekazu Kuramochi, Go Nakajima, Yuka Kaneko, Ayako Nakamura, Yuji Inoue, Masakazu Yamamoto, Kazuhiko Hayashi

**Affiliations:** 1 Department of Chemotherapy and Palliative Care, Tokyo Women's Medical University, 8-1 Kawadacho, Shinjukuku, Tokyo, Japan; 2 Department of Gastroenterological Surgery, Tokyo Women's Medical University, 8-1 Kawadacho, Shinjukuku, Tokyo, Japan

## Abstract

**Background:**

Amphiregulin (AREG) and Epiregulin (EREG), ligands of EGFR, are reported to be predictive biomarkers of colorectal cancer patients treated with Cetuximab, an anti-EGFR antibody. The purpose of this study is to determine the correlation of AREG and EREG expression between primary colorectal cancer and corresponding liver metastases.

**Methods:**

One hundred twenty colorectal cancer patients with liver metastases (100 with synchronous metastases, 20 with metachronous) were evaluated. No patients had ever received anti-EGFR antibody agents. AREG and EREG mRNA expression from both the primary tumor and liver metastases were measured using real-time RT-PCR. KRAS codon 12, 13 mutation status was analyzed by direct sequencing.

**Results:**

Modest, but significant, correlations were observed between primary tumor and corresponding liver metastases in both AREG mRNA expression (Rs = 0.54, p < 0.0001) and EREG mRNA expression (Rs = 0.58, p < 0.0001). AREG and EREG mRNA expression was strongly correlated in both the primary tumor (Rs = 0.81, p < 0.0001) and the liver metastases (Rs = 0.87, p < 0.0001). No significant survival difference was observed between low and high AREG or EREG patients when all 120 patients were analyzed. However, when divided by KRAS status, KRAS wild-type patients with low EREG mRNA levels in the primary site showed significantly better overall survival rates than those with high levels (p = 0.018). In multivariate analysis, low EREG expression was significantly associated with better overall survival (p = 0.006).

**Conclusions:**

AREG and EREG expression showed a modest correlation between primary tumor and liver metastases. As EREG mRNA expression was associated with decreased survival, it is appeared to be a useful prognostic marker in KRAS wild-type patients who never received anti-EGFR therapy.

## Background

Epidermal growth factor receptor (EGFR) is known to be involved in signaling pathways affecting cellular growth, differentiation, and proliferation [1]. To block the activation of this receptor, the anti-EGFR antibody agents Cetuximab and Panitumumab have been developed, and offer promising results for cases of metastatic colorectal cancer (CRC) [2-4]. Recently, several clinical trials demonstrated that somatic mutations in KRAS are associated with a lack of sensitivity to anti-EGFR antibody agents [5-7], suggesting that KRAS is a definite predictive biomarker for anti-EGFR antibody. However, even in patients with KRAS wild-type tumors, the response rates are between 10 and 40% [8]. Thus, other biomarkers are required for predicting which patients will benefit from anti-EGFR antibody therapy.

Amphiregulin (AREG) and Epiregulin (EREG) belong to the epidermal growth factor (EGF) family, and act as mitogenic stimulators through binding to EGFRs [9]. Recently, two large studies on AREG and EREG expression in patients with colorectal cancer who received Cetuximab were published. Khambata-Ford et al. analyzed the gene expression profile of 110 patients with colorectal cancer treated with Cetuximab to identify genes that were expressed differentially between the disease control group and the non-responders, demonstrated that AREG and EREG expression was associated with progression-free survival [10]. In this study, samples were collected from the metastatic site. Jacobs et al. examined 220 colorectal cancer patients treated with Cetuximab, and reported that there was a significant association between EREG and AREG expression and the response to Cetuximab in KRAS wild-type patients, but not in KRAS mutant patients [11]. Formalin-fixed paraffin-embedded (FFPE) samples from primary colorectal cancer were used in this study.

These two studies may indicate that AREG and EREG expression, as well as KRAS status acts as a predominant biomarker of sensitivity/resistance to Cetuximab. However, although the samples were taken from different sites in these two studies, there has been no previous study that has shown the relation between the gene expressions of AREG and of EREG between the primary site and a liver metastatic site. There has not as yet been clear that the gene expression from either primary or metastatic site is more associated with patients' prognosis. In addition, although these two independent studies showed the efficacy of AREG and EREG as predictive markers for Cetuximab, the clinical importance of these genes for patients who have not received anti-EGFR therapy is not clear. These genes are also reported to be associated with an increased likelihood of liver metastasis [12,13], which indicates that these genes may play an important role in the development of metastasis in malignancies, suggesting that they may be prognostic markers, even with patients who have never received anti-EGFR therapy.

Therefore, the aims of our study were: (1) to observe the relationship of AREG and EREG mRNA expressions between the primary site of colorectal cancer and the corresponding liver metastatic site, (2) determine whether the gene expression from the primary site or the liver metastatic site is more likely to be associated with clinical outcome, (3) determine the significance of AREG and EREG as a prognostic markers for patients who have not received anti-EGFR therapy, and see their relation to KRAS mutant status.

## Methods

### Patients and samples

One hundred and twenty cases of primary colorectal cancer with the corresponding liver metastases were analyzed in this study (70 men and 50 women; median age, 63.5 (range, 32- 91). The metastases in 100 patients were synchronous, and in 20 were metachronous. These patients had undergone surgical resection of primary colorectal adenocarcinoma and liver metastasis between 1998 and 2008 at the Department of Gastroenterology, Tokyo Women's Medical University, Tokyo, Japan. One hundred two patients had received fluoropyrimidinebased chemotherapy after surgery, 6 had never received chemotherapy and 12 were unknown. No patients had received neoadjuvant/adjuvant radiotherapy. No patients had ever received anti-EGFR antibody agents. All of the patients were Japanese, and all gave their written informed consent according to the institutional regulations. The characteristics of the 120 patients are shown in Table 1.

**Table 1:** Demographic and clinical parameters of patients with metastatic colorectal cancer

Characteristics	Frequency	%
Age		
Mean (range)	63.5 (32-91)	
Gender		
Male	70	58.3%
Female	50	41.7%
Anatomical Site		
Right colon	35	29.2%
Transverse colon	8	6.7%
Left colon	50	41.7%
Rectum	27	22.5%
Histology		
Well differentiated	89	74.2%
Moderately differentiated	27	22.5%
Poor/Mucinous	4	3.3%
Dukes Grade		
A	2	1.7%
B	34	28.3%
C	84	70.0%
Liver synchronicity		
Synchronous	100	83.3%
Metachronous	20	16.7%
Lymph node metastasis		
Positive	84	70.0%
Negative	36	30.0%
Adjuvant chemotherapy		
Received	102	85.0%
Not received	6	5.0%
Unknown	12	10%

This study was approved by the ethics committee of Tokyo Women's Medical University, and was performed in accordance with the Declaration of Helsinki.

### Microdissection

Formalin-fixed, paraffin-embedded tumor specimens were cut into serial sections with a thickness of 10 μm. For pathological diagnosis, one slide was stained with H&E and evaluated by a pathologist. Manual microdissection using a scalpel was performed if the histology was homogeneous and contained more than 90% of cancer cell tissue. For all other samples, laser-capture microdissection (P.A.L.M. Microlaser Technologies AG, Munich, Germany) was performed to ensure that only tumor cells were dissected.

### RNA isolation and cDNA synthesis

RNA isolation from formalin-fixed paraffin-embedded (FFPE) specimens was performed using an RNeasy FFPE Kit (Qiagen, Tokyo, Japan) according to the manufacturer's instructions. From the total RNA yielded, cDNA was converted using a High Capacity cDNA Reverse Transcription Kit (Applied Biosystems, Tokyo, Japan).

### Reverse transcription-PCR

cDNA was pre-amplified using a Taqman PreAmp Master Mix Kit (Applied Biosystems, Tokyo, Japan) according to the manufacturer's instructions. Quantification of AREG and EREG and internal reference gene (beta-2-microglobulin) was done using a fluorescence-based real-time detection method (StepOne real-time PCR system, Applied Biosystems Inc., Tokyo, Japan). Single internal reference gene (Beta-2-microglobulin) was used in this study. The primers and probes used were from Taqman Gene Expression Assays (Applied Biosystem Inc.), Assay IDs were Hs00950669_m1 for AREG, Hs00914312_m1 for EREG, and Hs99999907_m1 for beta-2-micoroglobulin. The PCR reaction mixture consisted of 10 μl of Taqman Fast Universal PCR Master Mix, No UNG (Applied Biosystem Inc.), 5 μl of preamplified cDNA sample, 1 μl of Taqman Gene Expression Assays primers and probe (20×), and 3 μl of Nuclease-Free Water. Cycling conditions were 95°C for 20 seconds, followed by 40 cycles at 95°C for 1 second and 60°C for 20 seconds. The threshold cycle (CT) value for each gene was determined by SDS software v1.2 (Applied Biosystems). Delta-CT(ΔCT) value, which is the difference between the CT value of the target gene and the CT value of the endogenous control gene was also calculated by the same software. Delta-ΔCT (ΔΔCT), which is the difference in the ΔCT value for each sample and the highest ΔCT value as a calibrator, was calculated. The 2^-ΔΔCT ^number was used for relative mRNA quantification.

Median values were used as the cut-off values to divide high and low expression.

### KRAS mutation screening

DNA was extracted from FFPE specimens using the Qiamp DNA FFPE tissue Kit (Qiagen, Tokyo, Japan) according to the manufacturer's instructions. Concentrations were measured with the ND-1000 Spectrophotometer (NanoDrop Technologies, Wilmington, DE, USA), and 500 μg of DNA was added to a 6 μl of forward and reverse primer and 25 μl of Quick Tag HS DyeMix (Toyobo, Osaka, Japan). Primers that spanned codons 12 and 13 of the KRAS gene were: forward, 5'--GAATGGTCCTGCACCAGTAA-3'; and reverse, 5'-GTGTGACATGTTCTAATATAGTCA-3'. PCR cycling was run according to the followingconditions: one cycle of 94°C for 3 minutes, 40 cycles of 94°C for 30 seconds, 56°C for 30 seconds, and 72°C for 45 seconds; and one cycle of 72°C for 10 minutes.

PCR products were purified using the MinElute PCR Purification Kit (Qiagen). The purified PCR product was then used as a template in cycle sequencing with the Dye Terminator Cycle Sequencing (DTCS) Quick Start Kit (Beckman Coulter, Tokyo, Japan) according to the manufacturer's instructions. Nested PCR primer sequences were Forward: 5'-GTCCTGCACCAGTAATATGC; reverse: 5'-ATGTTCTAATATAGTCACATTTTC-3'. Sequencing reactions were precipitated with ethanol, and run on a CEQ-8800 Genetic Analyzer (Beckman Coulter). Direct sequencing was performed in duplicate for each sample.

### Statistical analysis

The comparisons between the median mRNA levels of the primary tumor and the corresponding liver metastases, median mRNA levels of KRAS mutant and wild-type were assessed using the Wilcoxon signed-rank test. The correlation between the mRNA levels of primary tumor and of liver metastases was assessed using Spearman's rank correlation. The Kaplan-Meier method was used for survival curves, and the log-rank test was used for statistical analysis. Overall survival was defined as the time from the day of primary tumor resection to death from any cause. Multivariate analyses were performed by Cox proportional hazard model. Statistical significance was recognized at P-values of less than 0.05. All values were two-sided.

## Results

### AREG and EREG relative mRNA expression levels in primary colorectal cancer and liver metastases

There were no significant differences of median AREG and EREG relative mRNA levels between primary tumor and liver metastases (AREG: primary vs. liver = 0.16 vs. 0.21, p = 0.34, EREG: 0.030 vs. 0.034, p = 0.057) (Table 2).

**Table 2:** Median Values of AREG and EREG mRNA in primary tumor and liver metastases

	Primary tumor	Liver metastases	P-value
AREG (median)	0.16	0.21	0.34
(range)	(0.00-3.74)	(0.00-3.67)	N.S.
EREG (median)	0.030	0.034	0.057
(range)	(0.00-0.256)	(0.00-0.419)	N.S.

### Correlation of AREG and EREG expression between primary tumor and liver metastases

**Figure 1:**
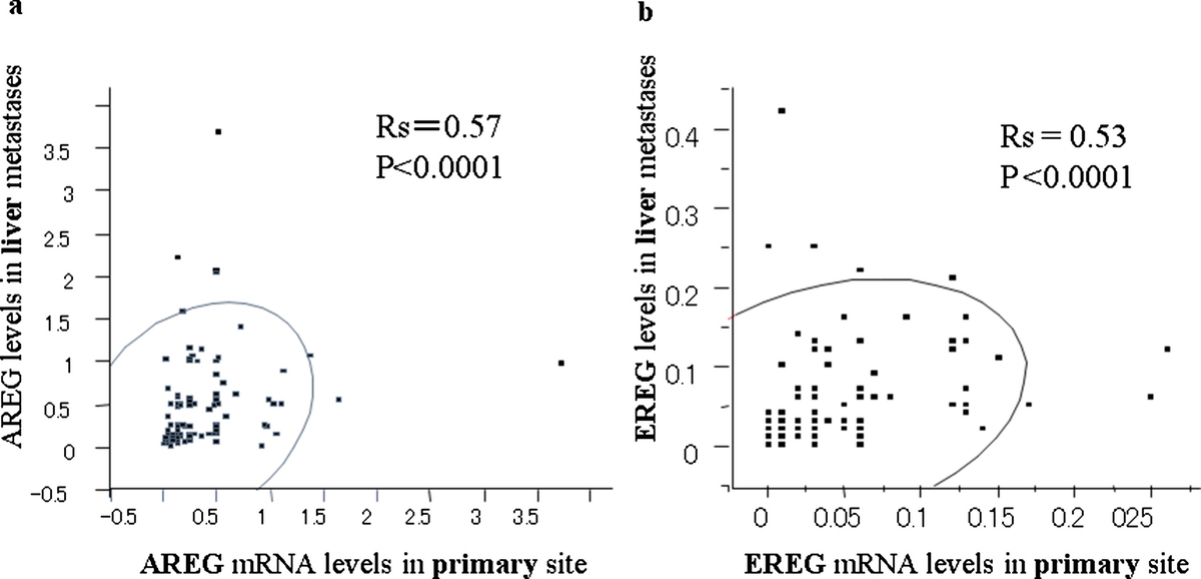
**Correlation of mRNA expression between primary colorectal cancer and corresponding liver metastases**. Figure 1-a: AREG mRNA expression; correlation coefficient (Rs) = 0.57, p < 0.0001. Figure 1-b: EREG mRNA expression; Rs = 0.53, p < 0.0001.

Modest, but significant correlation was seen with respect to AREG mRNA expression between primary tumor and corresponding liver metastases (Rs = 0.57, p < 0.0001) (Figure 1a). The same results were observed in EREG mRNA expression (Rs = 0.53, p < 0.0001) (Figure 1b). Regarding AREG expression, the correlation coefficient (Rs) was 0.357 in the patients with synchronous metastases, and 0.60 in those with metachronous metastases. Regarding EREG expression, the correlation coefficient (Rs) was 0.58 in the patients with synchronous metastases, and 0.36 in those with metachronous metastases.

### Correlation of expression levels between AREG and EREG

**Figure 2:**
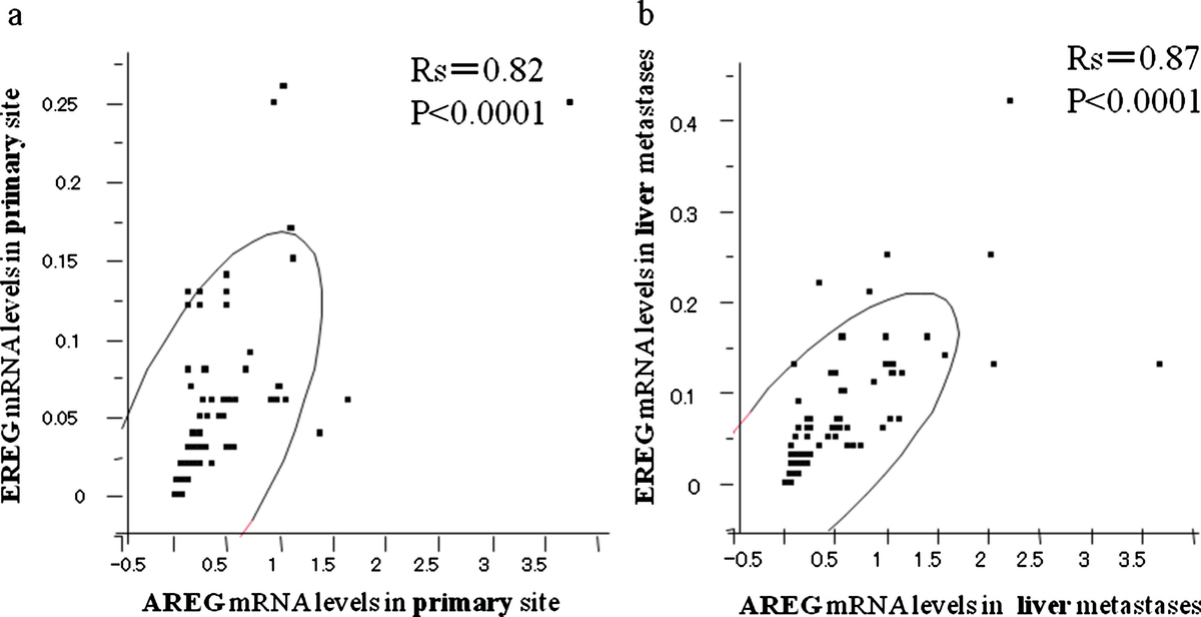
**Correlation of mRNA expression between AREG and EREG in primary colorectal cancer (2-a) and liver metastases (2-b)**. Figure 2-a: primary site; correlation coefficient (Rs) = 0.82, p < 0.0001. Figure 2-b: metastatic site; Rs = 0.87, p < 0.0001.

Strong correlations were observed between AREG mRNA expression and EREG mRNA expression in both the primary site (Rs = 0.82, p < 0.0001) (Figure 2a), and in the liver metastatic sites (Rs = 0.87, p < 0.0001) (Figure 2b).

### AREG and EREG expression and KRAS status

KRAS status was evaluated in 110 patients. Sixty-five (59%) patients showed the KRAS wild-type, and 45 (41%) showed the mutant type. No differences of median AREG or EREG mRNA levels were observed between KRAS wild-type and mutant types in the primary site (AREG: wild-type vs. mutant = 0.15 vs. 0.17; p = 0.40, EREG: wild-type vs. mutant = 0.027 vs 0.030; p = 0.45) or the liver metastatic site (AREG: 0.18 vs. 0.17; p = 0.86, EREG: 0.043 vs. 0.035; p = 0.73).

### AREG and EREG expression and overall survival by KRAS status

**Figure 3:**
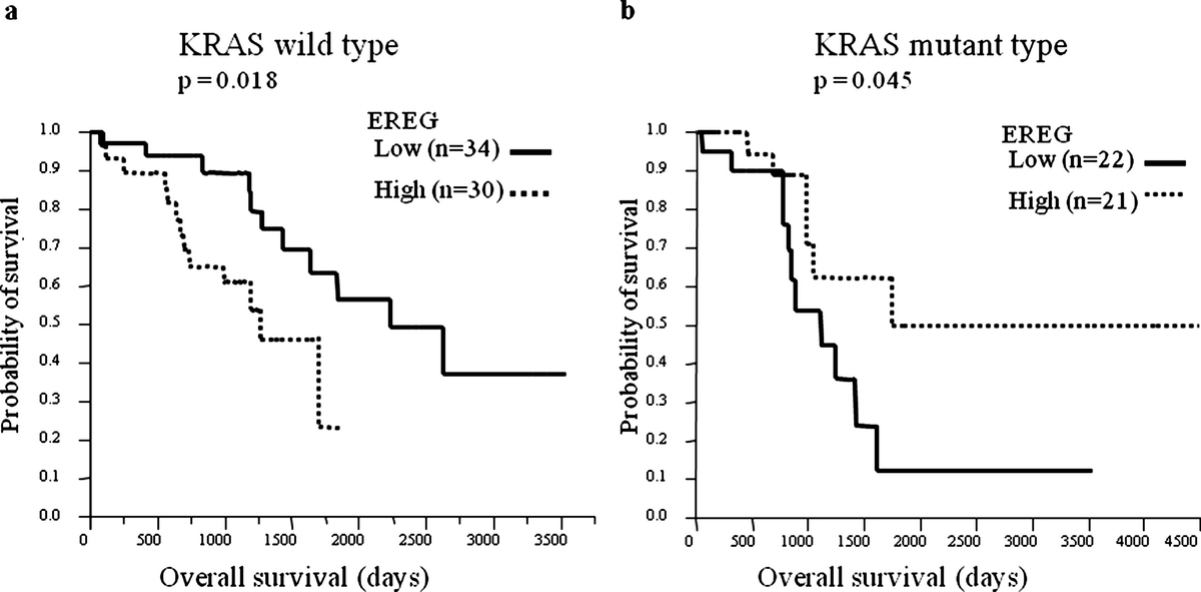
**Kaplan-Meier Curve of overall survival by EREG mRNA expression in KRAS wild-type group (3-a) and KRAS mutant group (3-b)**. Figure 3-a: KRAS wild-type group (p = 0.018, median survival; low vs high = 2222 vs 1190 days). Figure 3-b: KRAS mutant group (p = 0.045; median survival; low vs high = 1113 vs 1743 days). Cut-off line: median value of EREG expression levels.

There was no difference in overall survival between the patients with KRAS wild-type and those with mutant type (p = 0.71). When all 120 patients were analyzed, there were no survival differences between the patients with high AREG expression and those with low expression (p = 0.92), or between the patients with high EREG expression and those with low expression (p = 0.84) in the primary site. However, after division by KRAS status, the patients with low EREG expression in the primary tumor had significantly better overall survival than those with high expression in the KRAS wild-type group (p = 0.018, median survival time: 2222 days vs. 1190 days) (Figure 3a). The opposite result was observed in the KRAS mutant group; the patients with high EREG expression in primary tumor had better survival than those with low expression (p = 0.045, median survival time: high vs. low = 1743 days vs 1113 days) (Figure 3b). With respect to AREG expression in primary site, no statistical differences were observed in overall survival between the patients with low AREG expression and those with high expression either among the KRAS wild-type group (p = 0.16) or among the KRAS mutant group (p = 0.18). The relative mRNA expression levels in the liver metastatic sites did not correlate with overall survival time either among the KRAS wild-type group (AREG, p = 0.26; EREG: p = 0.28), or among the KRAS mutant group (AREG: p = 0.81, EREG: p = 0.63).

In multivariate analysis, low EREG expression in the primary tumor site was significantly related to better overall survival (p = 0.006) in KRAS wild-type patient group, as well as lymph node metastasis (p = 0.014) (Table 3)

**Table 3:** Multivariate analysis (Cox proportional hazard model)

KRAS Wild-type patients (n = 66)							
Factor	Cut-off	No. of pts	Median times (months)	Univariate analysis		Multivariate analysis	
				HR (95%CI)	p-valule	HR(95%CI)	p-value
EREG	Low	34	74.1	1.00	0.021*	1.00	0.006*
	High	30	42.1	2.80		3.73	
				(1.17-7.03)		(1.46-10.10)	
pN	Negative	15	not reached	1.00	0.022*	1.00	0.014*
	Positive	49	47.6	3.28		3.66	
				(1.17-11.74)		(1.28-13.34)	
Depth	T1,2	8	not reached	1.00	0.398	1.00	0.111
	T3,4	56	56.6	1.78		2.98	
				(0.52-11.16)		(0.80-19.41)	
**KRAS mutant patients (n **= **43)**							
Factor	Cut-off	No. of pts	Median times (months)	Univariate analysis		Multivariate analysis	
				HR (95%CI)	p-valule	HR(95%CI)	p-value
EREG	Low	22	37.1	1.00	0.048*	1.00	0.090
	High	21	58.1	0.37		0.42	
				(0.13-0.99)		(0.14-1.14)	
pN	Negative	17	not reached	1.00	0.182	1.00	0.383
	Positive	26	41.4	1.98		1.58	
				(0.73-6.25)		(0.58-5.03)	
Depth	T1,2	6	not reached	1.00	0.178	1.00	0.301
	T3,4	37	37.1	3.21		2.55	
				(0.65-58.11)		(0.51-46.40)	

## Discussion

In this study, modest correlations of AREG and EREG relative mRNA expression were observed between primary colorectal cancer and corresponding liver metastases. We have previously reported the correlation of VEGF [14], EGFR [15], and 5-FU metabolism-related genes [16] between primary colorectal tumor and liver metastases. Although EGFR mRNA expression showed a relatively strong correlation between the primary tumor and metastases [15], the correlations of AREG and EREG, which are the ligands of the EGFR family, between primary and metastases were weaker than that. The median values of AREG and EREG expression did not differ between primary cancer and metastases, which suggested that there was no up-regulation in the liver metastases. The strength of correlation was similar between synchronous and metachronous metastases, which suggested that expression levels were well preserved, even in relapse, for long time after primary tumor resection. Interestingly, the significance of EREG as a prognostic biomarker, which was observed in this study, was seen only on expression from the primary tumor, not on expression from the liver metastases. This data suggests that the expression from primary tumor reflects biological malignancy, and this relationship weakens in metastatic lesions after various biological modifications in the process of metastasis.

In this study, low EREG expression levels from primary tumors were significantly associated with overall survival only among patients with KRAS wild-type status. Although several previous reports demonstrated the association between EREG (and/or AREG) expression and survival time in KRAS wild-type patients who had received Cetuximab [10,11,17-19], no previous paper reported the association in colorectal cancer patients who had never received anti-EGFR antibody. The reason that this association was observed only in KRAS wild-type patients was probably that the regulation of this signal pathway depends mainly on the binding of ligands such as AREG and EREG to HER receptors in KRAS wild-type individuals, which suggests that higher expression of ligands results in up-regulation of this pathway and leads to cancer cell proliferation and the likelihood of metastasis. On the other hand, it is believed that KRAS mutations can lead to dysregulation of this pathway and downstream signaling in the absence of ligand-dependent receptor activation [10], and this suggested that the expression of ligands cannot be the main regulating factor of this pathway in KRAS mutant individuals. In our data, the patients with high EREG expression showed longer survival time in KRAS mutant patients (p = 0.045), in contrast to that in KRAS wild-type patients. However, since the numbers of patients with KRAS mutant were relatively small and the p-value was close to the significance level, this finding may not be reproducible, and probably needs to be validated.

Recently, several studies have reported that AREG (and EREG) expression was associated with the likelihood of liver metastasis in colorectal cancer, and suggested that it might play an important role in the development of liver metastasis [12,13]. In our study, a significant relationship was observed between EREG expression and patient survival, although all of the patients involved had liver metastases. This fact indicates that EREG and AREG expression is not only associated with the likelihood of metastasis but is also involved in tumor progression and biological malignancy. The association between these types of gene expression and survival time was also reported in other tumor types, such as non-small cell lung cancer [20] and oral squamous cell carcinoma [21]. These data suggested that EREG and AREG expression is not only a predictive biomarker for patients who have received anti-EGFR therapy, but is also a prognostic biomarker for a various types of cancer patients.

## Conclusions

In summary, this is the first study which showed the correlation of AREG and EREG mRNA expression between primary and corresponding liver metastases. This study also showed the significance of EREG mRNA expression as prognostic marker in patients with KRAS wild-type. These data appear to support the fact that this HER family signaling pathway plays a very important role in tumor progression, and blocking this pathway is a reasonable strategy for the treatment of colorectal cancer.

## Competing interests

The authors declare that they have no competing interests.

## Authors' contributions

HK conceived of the study, participated in the design of the study and drafted the manuscript. GN participated in the design of the study. YK carried out the DNA sequencing. AN performed the statistical analysis. YI participated in the design of the study. MY and KH helped to draft the manuscript. All authors read and approved the final manuscript.

## Pre-publication history

The pre-publication history for this paper can be accessed here:

http://www.biomedcentral.com/1471-2407/12/88/prepub
